# Primary cilia in myoblasts: a role in quiescence

**DOI:** 10.1186/2046-2530-4-S1-P79

**Published:** 2015-07-13

**Authors:** N Venugopal, J Dhawan

**Affiliations:** 1 Institute for Stem Cell Biology and Regenerative Medicine, Bangalore, India; 2 CSIR-Centre for Cellular and Molecular Biology, Hyderabad, India

## 

Quiescence and self-renewal are hallmarks of adult stem cells, which are essential for tissue homeostasis and regeneration. Earlier considered as a state of hibernation, G0 is now emerging as a balanced state where both the cell cycle and tissue-specific programs are held in check by active mechanisms. Quiescent cells are also marked by the presence of primary cilia, which are microtubule-based membrane-encased centrosome-derived structures that act as cellular antennae transducing chemical and mechanical signals. Here we investigate the role of the primary cilium as a determinant for cell cycle regulation and quiescence.

Using a mouse skeletal muscle myoblast culture system that can undergo either reversible or terminal arrest, we show that myoblasts show an increased propensity for ciliogenesis as they enter reversible arrest. By contrast when triggered to differentiate to multinucleated myotubes (irreversible arrest), cilia are lost after a transient phase of ciliation.

Blocking ciliogenesis by RNAi-mediated knockdown of ciliary transport protein IFT88 causes defects in cell cycle exit, with knockdown cells continuing to proliferate under conditions of quiescence. IFT88 knockdown cells also show a significantly lower clonogenic potential indicating that the self-renewal ability of cilium-ablated cells is compromised. Furthermore, transcriptional profiling by microarray revealed a strong signature of cell cycle related genes many of which are centrosome associated.

Our data suggests that primary cilia are important for the specific regulation of the program of reversible quiescence. We will present recent results that probe the function of primary cilia in quiescence.

